# Epidural anesthesia and analgesia in neonates and infants: Protocol for a scoping review

**DOI:** 10.1111/aas.70029

**Published:** 2025-04-20

**Authors:** Line Gry Larsen, Tom Geidsing Hansen

**Affiliations:** ^1^ Anestesiologic‐Intensive Ward V Odense University Hospital Odense Denmark; ^2^ Departpent of Anestesia and Intensive care Akershus University Hospital Nordbyhagen Norway

**Keywords:** epidural, infant, laparotomy, neonate, scoping, thoracotomy

## Abstract

**Background:**

Surgery and anesthesia in neonates and infants are performed only when necessary due to their immature physiological systems and limited cardiorespiratory reserves, rendering them particularly susceptible to complications. Effective pain management in the context of major surgical procedures presents a considerable challenge. Untreated pain activates cellular and humoral pathways that are detrimental to recovery. Although opioids are effective analgesics, their use is associated with hypoventilation and may exacerbate the physiological apnea commonly observed in newborns. This can extend the requirement for positive pressure ventilation, increasing the risks of both immediate and long‐term adverse outcomes. Epidural anesthesia and analgesia offer a promising alternative by providing effective pain relief while reducing opioid consumption, thus minimizing respiratory impairment and promoting more rapid recovery. This scoping review aims to map existing knowledge, to shed light on knowledge gaps, and lay the groundwork for further research.

**Methods:**

This review will follow the JBI methodology and adhere to the PRISMA guidelines for Scoping Reviews. A comprehensive search will be conducted across peer‐reviewed databases, published literature, online resources, ongoing studies, and gray literature to identify all available evidence on epidural anesthesia and analgesia in thoracic and abdominal surgeries in neonates and infants.

**Results:**

The findings from the included studies will be synthesized narratively and presented with tables and figures to ensure a clear and structured overview.

**Conclusion:**

This scoping review aims to summarize the current evidence on epidural anesthesia and analgesia in neonates and infants. It will highlight well‐researched areas, identify gaps in the literature, and outline existing knowledge and unmet research needs in this field.

## 1 INTRODUCTION

### 1.1 Background

Surgery and anesthesia in neonates and infants are performed only when necessary due to their immature physiology and limited physiological reserves. Cardiorespiratory reserves are significantly constrained: The circulatory system is challenged by (physiologic) shunts and fixed stroke volume with limited compensatory abilities, and the pulmonary alveolar ventilation surface area and functional residual capacity are markedly lower compared to adults.

Untreated pain triggers neurological, endocrine, and immunological pathways that are not conducive to recovery.^1^ While opioids are effective for managing pain, they compromise respiration and exacerbate the physiological apnea observed in neonates. Neonates also develop opioid tolerance rapidly, complicating the balance between opioid requirements, withdrawal symptoms, and effective pain management.^2^ Pain and changes in intrathoracic pressure can profoundly affect respiration, and thus potentially leads to atelectasis, hypercapnia, hypoxia, or even barotrauma resulting from positive pressure ventilation. For all the above reasons, an effective analgesic strategy is imperative for infants undergoing major surgical procedures, such as laparotomy or thoracotomy.

Epidural analgesia has emerged as a promising strategy, offering a favorable safety and efficacy profile without the risk of withdrawal symptoms or respiratory depression. It is a standard practice in our institutions; however, the extent of evidence supporting its use appears limited. From a global perspective, questions remain regarding the frequency of this practice, the procedures for which it is employed, the drugs and dosages used, and the associated adverse effects and complications. Some practitioners are reluctant to use epidurals in neonates and infants due to concerns about technical challenges, potential complications like hypotension or infection, and the risk of neurological complications, such as spinal cord injury or nerve damage, given their delicate anatomy.

This scoping review seeks to delineate the current state of knowledge in this field and identify gaps in the evidence. We aim to provide a foundation for future research by mapping existing knowledge.

## 2 METHODS

This scoping review will adhere to the JBI scoping review methodology,^3^, ^4^ and the Preferred Reporting Items for Systematic Review and Meta‐Analysis (PRISMA)^5^ for the Scoping Reviews checklist.

### 2.1 Registration

This scoping review protocol is registered in *Acta Anaesthesiologica Scandinavica* (AAS).

### 2.2 Eligibility criteria—Context

All study designs with human subjects will be considered, and no language restrictions will be applied in the primary search process. Studies of all neonates and infants (0‐ to 1‐year‐old) and from all countries, including low‐income ones, will be considered. Studies in languages other than English or Scandinavian will be processed by an online translation tool such as Google Translate and assessed for quality for further consideration.

### 2.3 Search strategy

The search will be performed in the following manner:

1. An initial limited search on MEDLINE, EMBASE, Scopus, CINAHL, and Scite, containing the keywords “epidural,” “neonates,” “infant,” “laparotomy,” “thoracotomy,” and their synonyms. This search will help uncover additional keywords, terms, and phrases for the next search phase. NOT sacral.
2. Specific searches with additional keywords/phrases uncovered in “1).”
3. A review of references found in the literature retrieved in “1)” and “2)” for the additional literature.

All papers retrieved from the above search strategy will be screened, and full texts will be retrieved. In the case of missing data according to our data charting, the authors of the papers will be requested to provide these if possible. Data missing will be noted as “unknown” or “not clear” in the charting. Papers excluded from further investigation will be explained.

TGH and LGL will independently participate in all screening and data extraction stages, addressing discrepancies through regular online meetings. If disagreements occur, resolution will be achieved by consensus or through the input of a third independent reviewer.

Gray literature^6^ will be screened. The search term “epidural,” will be used initially. If and only if any sources containing this term are found, the screening will progress.

We will look for “technical reports,” “white papers,” “policy briefs,” “working papers,” and “conference proceedings.”

We will search the following

- Google Scholar, OpenGrey, ProQuest, and Core
- Organizational homepages, including the World Health Organization (WHO), the European Society of Pediatric Anaesthesia (ESPA), and the Society of Pediatric Anesthesiologists (SPA)
- Research Gate
- The UK morphine infusion audit
- Direct contact with known experts within this field.

The final search strategy will be presented in the review paper. This will include at least one full search strategy from one of the databases, a description of additional search terms, phrases, and keywords, and an approach to gray literature. A flowchart from the PRISMA‐Scr checklist, presenting the search strategy, will be given.

### 2.4 Piloting

If the initial search using the keywords from “1)” retrieves >100 titles, we will perform “*pilot testing*” of 25 abstracts. We wish to agree on what sources to include in more than 75% of cases, leaving a maximum of 25% to a third reviewer. If a 75% agreement cannot be obtained, we will discuss the process further with this work and make relevant adjustments, such as revising keywords.

#### 2.4.1 Data management

The literature search results will be uploaded to CADIMA, a web‐based tool for streamlining and documenting the literature reviews. Duplicate entries will be systematically removed. Two authors will then independently evaluate all studies. In cases of disagreement, they will attempt to resolve the issue through discussion. If consensus cannot be reached, a third independent reviewer will be consulted, and their decision will be final.

## 3 RESULTS

### 3.1 Charting

All studies selected for inclusion will be charted by LGL, as in the predefined charts in Table 1. The charts might be modified as the search continues to include important knowledge revealed while uncovering the topic.

**TABLE 1:** Data charting form.

Title
Author(s)
Published date
Study type
Study Country
Population
Age
Gender
ASA score
Sample size
Surgery type
Main findings
Surgery procedure duration
Epidural procedure duration
Epidural technique
Level of insertion
Drugs, dosing and duration
Epidural satisfactory rate
Postoperative patient location
Length of hospital stay
Complications
Long‐term follow‐up
Cost and resources

### 3.2 Outcome measures

1. Time consumption for epidural installation and surgical procedure
2. Epidural technique and success rate, need for (additional) opioids, level of catheter placement
3. Drugs, dosing, and duration
4. Postoperative patient location and admission time
5. Safety and complications: Toxicity, accidental spinal access, hematomas
6. Satisfaction: Staff, parents, patient
7. Cost and resources
8. Long‐term follow‐up

#### 3.2.1 Data synthesis and presentation

The collected data will be presented using the specified charts. Additionally, a graphical representation tailored to the nature and volume of the search results will be created.

#### 3.2.2 Confidence in the retrieved evidence

A systematic evidence synthesis will not be performed. Instead, the findings will be summarized narratively.

## 4 DISCUSSION

This review aims to consolidate existing knowledge on epidural anesthesia in newborns and infants, providing a basis for future research in this field. Epidural anesthesia shows significant promise as an effective approach to pain management in this vulnerable population. Reports suggest that, in some cases, infants even spontaneously fall asleep following the insertion of the epidural catheter without the need for general anesthesia.^2^

Epidural catheter insertion has been described as straightforward and safe in resource‐limited settings and tertiary hospitals with limited access to advanced airway management expertise for neonates^7^.

The reasons for adopting or avoiding epidural anesthesia remain unclear, and this review seeks to explore these factors. Questions surrounding global perceptions of epidural safety, its prevalence of use, and the specific drugs and dosages employed remain unanswered. We hope this review will illuminate these aspects and contribute to a more comprehensive understanding of the practice.

## AUTHOR CONTRIBUTIONS

Tom G. Hansen provided the subject and content of this protocol, which Line Gry Larsen structured. Line Gry Larsen will do the forthcoming data charting and graphical presentation, while Tom G. Hansen and Line Gry Larsen will assess the data in collaboration, regularly discussing findings and reasonable means of presentation.

## FUNDING INFORMATION

Tom G. Hansen and Line Gry Larsen did not receive any funding for this work.

## CONFLICT OF INTEREST STATEMENT

Tom G. Hansen is a professor at Akershus Universitetssykehus, a pediatric anesthesiologist and an associate Editor of *Acta Anaesthesiologica Scandinavica*.

Line Gry Larsen is a pediatric anesthesiologist from Odense University Hospital and declares no conflict of interest.

## Data Availability

Data sharing does not apply for this article as no datasets were generated or analyzed during the generation of this study protocol.
